# Evaluation of a multi-component early warning system for pastoralist populations in Doolo zone, Ethiopia: mixed-methods study

**DOI:** 10.1186/s13031-024-00571-y

**Published:** 2024-01-30

**Authors:** Luke Baertlein, Bashir Ali Dubad, Birhanu Sahelie, Istifanus Chindong Damulak, Mohammed Osman, Beverley Stringer, Agatha Bestman, Anna Kuehne, Elburg van Boetzelaer, Patrick Keating

**Affiliations:** 1 Médecins Sans Frontières, Addis Ababa, Ethiopia; 2 Somali Regional Health Bureau, Jigjiga, Ethiopia; 3 Médecins Sans Frontières, London, UK; 4 Médecins Sans Frontières, Nairobi, Kenya; 5 London School of Hygiene and Tropical Medicine, London, UK

**Keywords:** Surveillance, Pastoralist, Evaluation, Early warning, Ethiopia

## Abstract

**Background:**

This study evaluated an early warning, alert and response system for a crisis-affected population in Doolo zone, Somali Region, Ethiopia, in 2019–2021, with a history of epidemics of outbreak-prone diseases. To adequately cover an area populated by a semi-nomadic pastoralist, or livestock herding, population with sparse access to healthcare facilities, the surveillance system included four components: health facility indicator-based surveillance, community indicator- and event-based surveillance, and alerts from other actors in the area. This evaluation described the usefulness, acceptability, completeness, timeliness, positive predictive value, and representativeness of these components.

**Methods:**

We carried out a mixed-methods study retrospectively analysing data from the surveillance system February 2019–January 2021 along with key informant interviews with system implementers, and focus group discussions with local communities. Transcripts were analyzed using a mixed deductive and inductive approach. Surveillance quality indicators assessed included completeness, timeliness, and positive predictive value, among others.

**Results:**

1010 signals were analysed; these resulted in 168 verified events, 58 alerts, and 29 responses. Most of the alerts (46/58) and responses (22/29) were initiated through the community event-based branch of the surveillance system. In comparison, one alert and one response was initiated via the community indicator-based branch. Positive predictive value of signals received was about 6%. About 80% of signals were verified within 24 h of reports, and 40% were risk assessed within 48 h. System responses included new mobile clinic sites, measles vaccination catch-ups, and water and sanitation-related interventions. Focus group discussions emphasized that responses generated were an expected return by participant communities for their role in data collection and reporting. Participant communities found the system acceptable when it led to the responses they expected. Some event types, such as those around animal health, led to the community’s response expectations not being met.

**Conclusions:**

Event-based surveillance can produce useful data for localized public health action for pastoralist populations. Improvements could include greater community involvement in the system design and potentially incorporating One Health approaches.

**Supplementary Information:**

The online version contains supplementary material available at 10.1186/s13031-024-00571-y.

## Background

Médecins Sans Frontières (MSF) has provided medical assistance in the Doolo zone, Somali region Ethiopia since 2007, with the initial aim of providing primary and secondary healthcare to conflict-affected populations. With unrest ending in 2014, MSF continued to support increasing access to healthcare. A complex emergency subsequently developed in 2017, when failed seasonal rains led to widespread drought and livestock deaths, a nutrition crises and disease outbreaks, including acute watery diarrhea (AWD), measles, and acute jaundice syndrome (AJS) [1]. In response, MSF launched emergency interventions, including nutrition, measles vaccination, and cholera case management activities, continuing until mid-2018. When the crisis subsided, MSF identified the need to develop systems to rapidly identify and respond to public health hazards. A community indicator-based surveillance strategy was first introduced in 2017, and was modified in 2019 into an early warning, alert and response (EWAR) system with four components: (1) health facility indicator-based surveillance, (2) community indicator-based surveillance, (3) community event-based surveillance and (4) event-based surveillance using alerts from other actors (e.g. Doolo zone’s Regional Health Bureau (RHB) [2]. This multi-component approach was chosen given high population mobility and limited access to care in the community, as well as pre-existing surveillance activities. The system was informally referred to as the “Tea Team surveillance system” since some surveillance data were collected via exchanges in traditional tea rooms. These tea rooms are social gathering points in the community, inclusive of both the settled population and pastoralist semi-nomadic groups. The community event-based surveillance component involved community discussions on what priority health events should be included in the system, as well as discussion around data collection processes that key informants would use to alert MSF of potential public health emergencies [3]. This multi-component approach to surveillance ensured coverage of communities with and without access to healthcare as well as covering nomadic and settled communities, with the objective of detecting all events with potential public health risk in a timely manner, enabling rapid risk assessment and appropriate response [4].

There have been few evaluations of such multi-component surveillance systems with event and indicator components, based in both community and health facilities, although integration of event-based surveillance with other early warning and response systems has been noted as an important factor [5]. The objective of our study was to evaluate the following attributes of the surveillance system: usefulness, acceptability, completeness, timeliness, positive predictive value, and representativeness.

## Methods

### Study setting

Doolo zone is one of nine zones in the Somali region of Ethiopia. Based on Ethiopia’s 2007 census, the zone’s total population is 306,488, of whom 42.7% were women [6]. Estimated population for 2019 is 556,870, with 37% of the zone population thought to be pastoralist. During our evaluation MSF ran 15 mobile clinics across the zone, from which health and community indicator-based surveillance and community event-based surveillance data were collected. In addition, there were 17 further locations where community event-based surveillance data were collected for a total of 32 locations within the Tea Team surveillance system.

### Study design

We used a mixed-methods approach including retrospective analysis of data captured by the surveillance system, key informant interviews (KIIs) with MSF implementers, and focus group discussions (FGDs) with engaged communities, to describe the system as it was implemented and to evaluate it.

### Retrospective analysis of surveillance data

Analysis included data from each of the four surveillance components, which were included in a central MSF EWAR database covering February 2019 through January 2021 (Table 1 for definitions).

**Table 1:** Definitions of data included in MSF’s EWAR database, based on WHO EWAR definitions [2]

Term	Definition
Signal	A signal is reported information, for example rumours, or information that represents a potential acute risk to human health. It has not yet been verified as to whether or not it meets the event definition of the surveillance system
Event	Reported information (signal) on a potential acute risk to human health that has been verified
Alert	A public health event that has been (i) verified and (ii) risk-assessed and (iii) requires a response (an investigation, an intervention or a communication)
Response	Any public health action triggered by the detection of an alert, such as vaccination and water, sanitation and hygiene (WASH)-related activities, among others

Health facility indicator-based surveillance (HFIBS) data were reported in the format of weekly aggregate counts of consultations by disease category for each mobile clinic. In addition, any emergency signals from locations where there were mobile clinics were intended to be captured through event-based surveillance. Unfortunately, signals from HFIBS were not consistently added to the MSF EWAR database and thus, this branch of the surveillance system was not included in the evaluation.

For community indicator-based surveillance (CIBS), weekly counts of specific diseases and health events occurring in a community were collected and reported by community health workers. These were analysed to identify signals based on CIBS signal criteria.

For community event-based surveillance (CEBS), community informants were selected and trained to report signals to MSF either through a weekly scheduled phone call or through an emergency call. Signals reported through the scheduled phone call were first recorded in a weekly call log separate from the MSF EWAR database and later transferred into the MSF EWAR database if they met pre-verification criteria, as assessed by health education supervisors familiar with the communities. The pre-verification process removed signals that did not meet signal definitions and were clearly not health events, to avoid an excessive amount of signals requiring more formal verification.

In addition, signals could be gathered at bimonthly meetings at tea sessions with community members; these would be entered into a bimonthly meeting form and later the MSF EWAR database, as above. Emergency signals were recorded directly into the MSF EWAR database.

For other event-based surveillance (OEBS), signals reported by other actors such as the RHB and non-governmental organizations working in the area were recorded directly into the MSF EWAR database once received.

For signals that underwent verification, assessment, and response, signals were added to the EWAR database, including information on subsequent verification and assessment findings as well as on what response was undertaken. Neither the specific signal nor the system that was the source of the signal (i.e., HFIBS, CEBS, CIBS, or OEBS) were recorded in the MSF EWAR database, creating difficulties in mapping received signals to those that were recorded in the MSF EWAR database. To enable assessment of verification, assessment, and response outcomes for signals reported through each of the different surveillance components, the signal data were matched to the MSF EWAR database by week reported, originating community name, and type of disease or health event signalled. The matched dataset was then analysed to describe a set of quantitative indicators of surveillance system performance for signals originating from each of the four surveillance components [7, 8] (Table 2). Without the true number of outbreaks, we were unable to calculate sensitivity.

**Table 2:** Indicators per surveillance system attribute

Attribute	Indicator
Usefulness	Number of alerts that resulted in public health action
	Type of response resulting from alerts
	Proportion of alerts reported through each source
Completeness and consistency of reporting	Completeness of information in the EWAR cascade
Positive predictive value	Proportion of events, alerts, and responses identified out of the total number of signals received
Timeliness of signals, assessment, and response	Proportion of signals verified within 24 and events assessed within 48 h after reporting
	Median time to assessment and response after reporting

All quantitative analyses of data from the community indicator- and event-based and other event-based surveillance systems were conducted using R software [9].

### KIIs with MSF staff

Individual KIIs were held to evaluate staff perceptions regarding three evaluation domains; usefulness, representativeness, and acceptability. A total of eight staff were interviewed using a semi-structured format. Staff were selected purposively to ensure each of the main roles in the surveillance system were represented. This included the epidemiologist, the health promotion and community education activity manager, the data encoder, two nursing team supervisors, two health education supervisors and a mobile clinic nurse. Interviews were conducted in English and transcribed from audio recordings. Interview transcripts were analysed using a mixed deductive and inductive approach. Deductive themes were generated from the study protocol (e.g., surveillance attributes) and from review of key project documents. Inductive themes were generated through review of the transcripts and then applied consistently across all transcripts. Transcripts were reviewed and coded and the codes were grouped under pre-identified themes using a framework approach to facilitate qualitative data synthesis and exploration of patterns across and within transcripts.

### FGDs with communities

In order to understand community perspectives on the surveillance system’s usefulness, representativeness, and acceptability, FGDs were held with eight communities, of which four participated in the Tea Team package of services (CEBS, response, and routine health promotion); four participated in the mobile clinic package (HFIBS, CIBS and mobile clinic services). Communities were selected for participation based on convenience and current participation in surveillance. Participants in each community were selected so that each discussion group included representatives of key roles, comprising local informants (Tea Team sites) or Community Health Workers (Mobile Clinic sites), local leaders or chairmen, elders, and women. The discussion groups had 6–8 participants.

FGDs were facilitated by two trained and experienced facilitators—one moderator and one note-taker. Discussions were audio-recorded and all discussions took place in Somali. After discussions were completed, audio recordings and notes were used to prepare verbatim note transcripts in Somali, which were translated into English. The English translations were analyzed using a mixed deductive and inductive approach. Deductive and inductive themes were generated as above for KIIs. All qualitative data analyses were performed with Lumivero (2018), *NVivo* (Version 12), www.lumivero.com [10].

## Results

### Description of the surveillance system

**Fig. 1:**
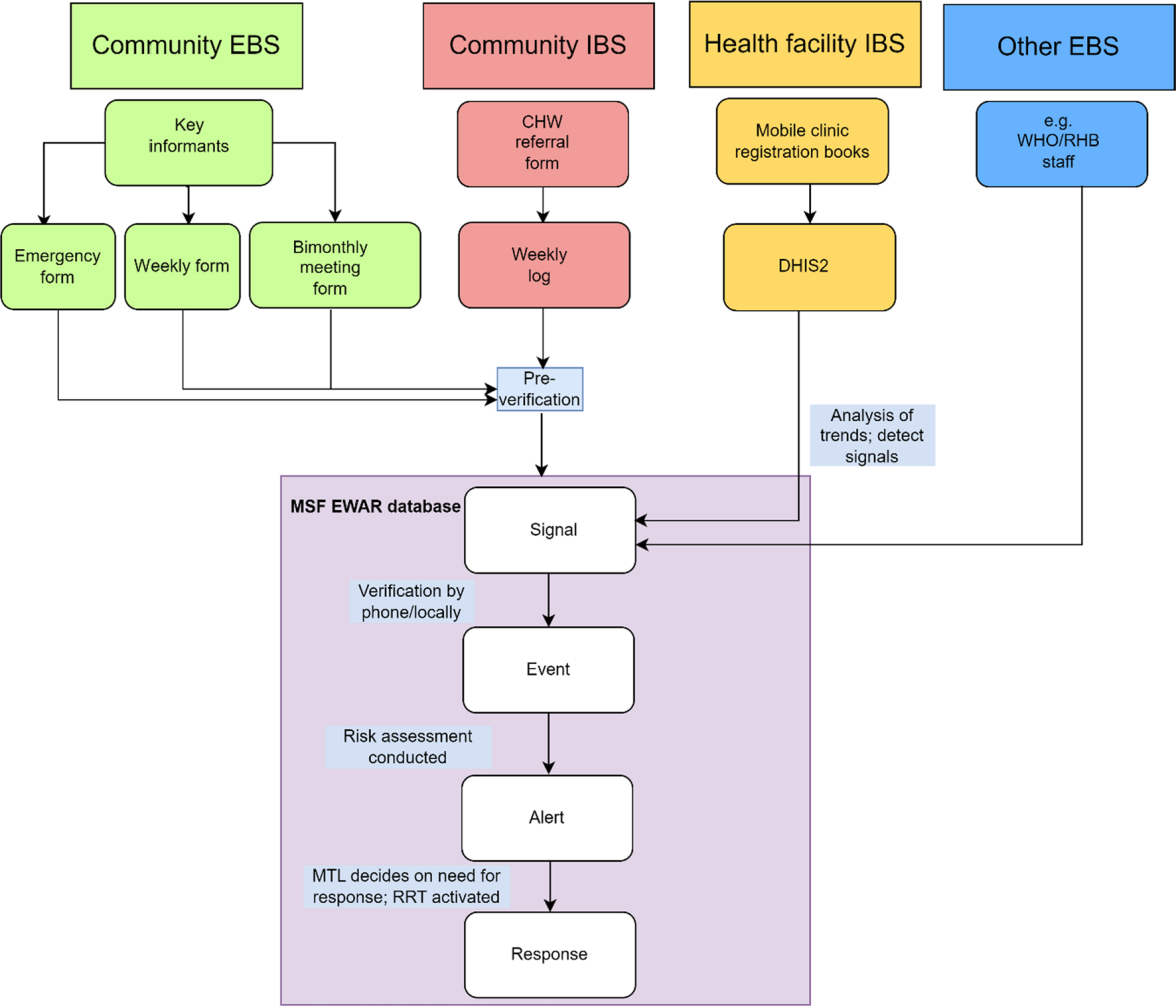
Data flow in the Tea Team surveillance system. *MTL: Medical Team Leader

The Tea Team surveillance strategy was designed to integrate with the available health facility network as well as to cover communities without access to health facilities. This was done through a four-component surveillance approach, each of which fed into a centralized process of verification, risk assessment, and response (Fig. 1). An additional table describes the roles and responsibilities in detail [see Additional file 1].

*Community event based surveillance (CEBS)* MSF implemented CEBS in communities with partly-functional RHB-operated health facilities. In these communities, MSF collected signals from community members and local informants. Informants were selected through engagement with community leaders and members, based on their willingness and community acceptance.

Based on community discussions, a set of priority health events were agreed upon and volunteer community informants were provided with instructions on how to identify and report these events, in addition to any other unusual health event. These included: suspected cases or clusters of cases of AWD, measles and AJS, increased number of deaths in the community, malnutrition, mass animal sickness or death, and population movements. CEBS signal and event definitions for each of these are provided in more detail in an additional table [see Additional file 1].

Feedback phone calls to informants following reporting of a signal were used to reinforce knowledge of signal criteria, complemented by monthly supportive supervision during visits by MSF staff to the community.

*Community indicator-based surveillance (CIBS)* In communities without RHB health facilities that had MSF mobile clinics, the mobile clinic program included community health workers recruited as MSF staff from each community served by a mobile clinic. Responsibilities of community health workers included providing health education to the population and sharing information about clinic services to the community, through routine household visits in the community. Each household was intended to be visited once per month. These household visits organized by the mobile clinic program to make referrals to the mobile clinic or other healthcare services when necessary, and were leveraged by the CIBS system to identify and report cases of suspected AWD, measles, AJS and malnutrition CIBS signal definitions are available in an additional table [see Additional file 1].

CHWs were also asked to report signals through the CEBS emergency form, when identified, to facilitate more timely reporting than CIBS.

*Other event-based surveillance (OEBS)* The final component of the Tea Team surveillance system was also passive. To detect health events occurring outside of CEBS and CIBS/HFIBS communities, the activities of other agencies in Doolo zone were relied upon. If signals under MSF surveillance were picked up informally or through their system, they shared the signal information with MSF through an informal communication network. MSF teams then verified, assessed, and responded. This informal gathering of signal information from partner agencies is referred to as OEBS.

*Signal verification* Firstly, there was a process of pre-verification: only signals that fulfilled pre-determined criteria were added to the MSF EWAR database. The criteria varied by disease type and were related to the number of cases, symptoms, or clustering of cases. Signals that did not pass this pre-verification were discarded. Once a signal passed pre-verification and was added to the MSF EWAR database, the process of verification of the signal as an event, assessment of an event as an alert, and response to alerts was started.

For all signals added to the database, from all sources, the epidemiologist used case definitions and thresholds for verification purposes/to decide on whether it constituted an event (and assessment was needed), whether it was a ‘false signal’ (no further action is needed), or whether more information gathering was needed before a decision can be taken. If more information was needed, the epidemiologist collected this through phone calls to community informants and speaking to community outreach staff and volunteers.

*Risk assessment* When a signal was verified as an event, an on-site assessment was conducted by a rapid response team (RRT) consisting of the epidemiologist, health educators, medical staff, and water/sanitation staff. The assessment included visits to any health facility serving the affected community, 5–10 households around the suspected cases, and to community elders. This visit also typically included reactive response among the immediately affected households such as distribution of chlorine sachets and soap. The information gathered during the assessment was then analysed and used to inform decisions on what response by MSF was needed, if any, and to inform response planning.

*Response* The types of alerts to which MSF responded was set periodically using an emergency preparedness plan. This plan specified both the types of diseases MSF would have the capacity to respond to, the thresholds for response for each disease, and an outline of what the response would entail. Responses typically entailed WASH (e.g. chlorination, hygiene kit distribution), medical case management, vaccination, enhanced surveillance, and health education, tailored to the alert type and context. Responses to population movements included re-location of mobile clinic teams providing primary healthcare services.

### Summary of surveillance data

In total, 1010 signals were received by the Tea Team surveillance system via its CEBS, CIBS and OEBS components between February 2019 and January 2021. Of these, 265 (26%) passed pre-verification and were added to the EWAR database (Table 3). Out of 265 signals added to the EWAR database, 168 (63%) were verified as events and 119 (71%, 119/168) of those were risk-assessed. Fifty-eight (49%, 58/119) of the risk-assessed signals became alerts and 29 (50%, 29/58) alerts were responded to by MSF.

**Table 3:** Signals, events, alerts, and responses by surveillance type, February 2019 through January 2021

Surveillance Type	Total signals received	Signals in EWAR database	Verified events	Alerts	Responses
CEBS	916 (91%)	199 (75%)	129 (77%)	46 (79%)	22 (76%)
OEBS	62 (6%)	62 (23%)	37 (22%)	11 (19%)	6 (21%)
CIBS	32 (3%)	4 (2%)	2 (1%)	1 (2%)	1 (3%)
Total	1,010 (100%)	265 (100%)	168 (100%)	58 (100%)	29 (100%)

### Usefulness

During the study period, the system received 58 alerts, of which 29 received a response. Among the 58 alerts, 46 were triggered by CEBS, 11 by OEBS, and 1 by CIBS (Table 4). Most alerts were population movements (15), followed by suspected AWD (14) and suspected measles (11).

**Table 4:** Response to alerts by surveillance type of signal and signal class

Signal Class	Type of surveillance	Total alerts reported	Responses	Percent of alerts responded to
Total	CEBS	46	22	48%
	OEBS	11	6	55%
	CIBS	1	1	100%
Suspected AWD/Cholera	CEBS	12	6	50%
	OEBS	1	0	0%
	CIBS	1	1	100%
Suspected Measles	CEBS	7	5	71%
	OEBS	4	2	50%
Food Insecurity/ Malnutrition	CEBS	3	0	0%
Population movement	CEBS	13	8	62%
	OEBS	2	2	100%
Other^1^	CEBS	11	3	27%
	OEBS	4	2	50%

^1^Other includes Covid-19, water shortage, lack of healthcare, and flooding

Out of all responses conducted, 22 (76%) originated from signals picked up by CEBS, six (21%) from OEBS, and one (3%) from CIBS. Among response types, there were 10 for population movements, seven for suspected measles, seven for suspected AWD, three for flooding, and one each for water shortage and lack of health care service. The common responses launched during the evaluation period include starting new mobile clinic sites (for population movements), doing measles vaccination catch-ups, and WASH responses. Many responses included multiple intervention types and were reinforced by targeted health promotion messaging.

Likelihood of system response aligned with views around the primary purpose of the system. This is similar to community responses in FGD’s, where response was described as an expected outcome, in return for their participation in data collection and reporting.

*“It is important for us when there is a benefit, or there is a risk of harm to us, so it is important for the community to pass on what is available to those who are concerned so that they can respond to any benefit and there is nothing important for the community if they do not get any response for the grievances they presented, what the community is interested in is what they gain and lose.” (Community FGD participant)*

### Completeness

In the overall system, 26% of reported signals were recorded in the EWAR database, among which 76% were complete in the EWAR cascade (i.e. not left as a pending) (Table 5).

**Table 5:** Signal follow-up completeness by surveillance type

Surveillance type	Total number of signals	Number of Signals in EWAR database after pre-verification	Number of Signals with follow-up completed^1^	Percent of signals entered into EWAR database after pre-verification	Percent of signals with follow-up completed, of total	Percent of signals with follow-up completed, of those entered into EWAR database
CEBS	916	199	150	22	16	75
OEBS	62	62	48	100	77	77
CIBS	32	4	3	13	9	75
Total	1010	265	201	26	20	76

^1^Follow-up completed means that a signal went through all relevant processing steps from verification, risk assessment and response, as appropriate

### Positive predictive value

Signals reported via OEBS had a higher positive predictive value (PPV) than CEBS (Table 6). Suspected measles had the highest event PPV compared to all other signals reported Suspected measles and suspected AWD had the highest alert PPVs.

**Table 6:** Event and alert PPV by surveillance type and signal class

Signal class	Surveillance type	Total signals reported	Total events	Total alerts	Event PPV of a reported signal (%)	Alert PPV of a reported signal (%)
Total	CEBS	916	129	46	14	5
	OEBS	62	37	11	60	18
	CIBS	32	2	1	6	3
Suspected measles	CEBS	54	26	7	48	13
	OEBS	20	15	4	75	20
	CIBS	7	1	0	14	0
Suspected AWD	CEBS	75	21	12	28	16
	OEBS	9	3	1	33	11
	CIBS	21	1	1	5	5
Suspected AJS	CEBS	17	9	0	53	0
	OEBS	2	1	0	50	0
	CIBS	4	0	0	0	0
Deaths	CEBS	150	1	0	1	0
	OEBS	1	0	0	0	0
Malnutrition	CEBS	137	6	3	4	2
	OEBS	4	1	0	25	0
Other concerns	CEBS	483	66	24	14	5
	OEBS	26	17	6	65	23

### Timeliness

Timeliness was related to the utility of the surveillance system in guiding response. Timeliness in detection and response was seen as important in enabling response before an outbreak might grow beyond the scope of the project’s response capacity.

*“It is important early detection and response before it goes beyond our capacity.” (MSF staff interviewee)*

Out of all signals verified across the four components, 81% of them were verified within 24 h of reporting (Table 7). Overall, the timeliness of verification, risk assessment, and response following a signal being reported was similar across surveillance types.

**Table 7:** Timeliness of verification, risk assessment, and response of reported signals added to the EWAR database

Surveillance type	Percent of signals verified within 24 h of report	Percent of events risk assessed within 48 h of report	Median days from report to response (IQR)
CEBS	81% (148/183)	38% (36/95)	9 (2, 14)
OEBS	80% (47/59)	46% (12/26)	6 (3, 19)
CIBS	75% (3/4)	100% (1/1)	1 (1, 1)

### Representativeness

Geographic representativeness of public health hazards identified in the Doolo zone was closely related to coverage, which was limited to 32 communities. Surveillance at these sites was mostly via CEBS and additional coverage was sought via the other event-based surveillance component.

The Tea Team surveillance system was more representative than a system where event types were rigidly defined, in that it could capture many types of events that were not pre-specified, e.g., water, food supply, or livestock health issues.

In terms of community’s reporting, data representativeness was described as being impacted by the number of trained informants or community health workers (CHWs) in a community. In geographically disparate communities or those with large, dispersed pastoralist populations, FGDs indicated that more informants or CHWs would be needed to adequately collect health event information in order to improve completeness and representativeness.

*“We are also advising the agency to increase our workforce. The population is very large, and it is nomadic. […] We have three villages that are connected and the people are many, nomadic people are very many.” (Community FGD participant)*

In addition, FGD participants stressed that training women in identifying health events and reporting them was essential to improving completeness and representativeness. FGD participants commented that the information available to women is different than that to men, with women having better access to information about events impacting mothers and children than men.

*“Women will not tell everything to men workers, as women from the countryside are very shy, therefore, for women to report their needs and health concerns, increase the female workers. […] We are requesting female members be increased because women are more vulnerable and in order to have someone whom women can submit their views to.” (Community FGD participant)*

### Acceptability

Many FGD participants described the Tea Teams as a system through which the community could request assistance for specific health events. A response was expected for each event reported, and lack of timely response or feedback on every reported signal or event led to lower acceptability of the system among the community participants due to unmet expectations.

*“The unhealthy issues of the camels have been reported and it was not responded to. The expectation we had from the unhealthy issues of the camels is not yet met. I think you expected that these are inspected and responded to immediately but this has not happened. Mostly the issues are not responded to on time.” (Community FGD participant)*

Similarly, when cases of illness were reported, specific response to assist reported cases was expected, in addition to population-level responses such as those aimed at preventing further spread of disease. For instance, if individual measles cases reported required referral to higher-level healthcare for complications, it was expected that MSF would facilitate referrals, in addition to conducting vaccination catch-up campaigns to prevent further spread. When only population-level or prevention-oriented interventions were delivered, the system was seen as less acceptable.

*“Such incidents responded to by MSF include: there were people who were burned by fire and the agency took them and treated them well, also there were women who were in labor and the agency took them to a health center where some gave birth through operation and the others were given medicine by the agency and got well." (Community FGD participant).*

## Discussion

We found that Tea Team surveillance effectively detected and responded to public health events and met the primary objective of producing useful data for public health action. Strengths were identified in terms of the complementary use of different sources of information and the partly participatory surveillance activities conducted, while weaknesses included the lack of focus on animal health, the lack of a clear community feedback mechanism and limited representativeness.

A key strength of this system was that MSF staff gathered information on hazards through existing discussion spaces, e.g. tea rooms, enabling capture of alerts from pastoralist and village members. This approach was similar to that taken by CHWs involved in acute flaccid paralysis surveillance in Ethiopia where traditional coffee ceremonies provided a venue [14]. Nevertheless, alongside holding these discussions in tea rooms, MSF could have further engaged within other community organizations and decision-making structures [15]. This approach would align better with core humanitarian standards and views on the localisation of aid [16, 17]. Such approaches might improve expectation-setting and overall acceptability, as these would mean MSF teams would interact consistently with key decision makers within their preferred contexts. Communication and community engagement have been identified as key success factors for community based surveillance (CBS) and particularly in relation to clear and consistent community feedback, which the Tea team surveillance system was lacking [2, 13]. This issue could have been addressed at design phase and ultimately might improve overall system functioning and acceptability.

The inclusion of a partly participatory surveillance component (discussion on priority health events and data collection processes) via the CEBS branch was an additional strength of this system [3]. Participatory surveillance components have been used in other CBS implementations to improve timeliness of outbreak detection [15, 18, 19]. Telephone-based participatory data collection methods have been used to report animal and human health events in the Somali region; these were able to rapidly detect animal and human-related outbreaks [15].

Animals are a key source of wealth of pastoralist communities and ultimately animal and human health are interconnected, such that death and illness of livestock impacts on human health. This was clearly demonstrated by failed rains and sequelae of this in the Doolo zone in 2017–2018 emergency period. A key limitation of the system was the lack of a One Health approach where human and animal health sectors collaborate in outbreak detection and response. This deficiency impacted system acceptability, as animal health issues were reported by the population, but MSF had no capacity to act on them directly. Previous One Health surveillance in the Somali region in 2017/2018 detected suspected outbreaks in animals (e.g. anthrax) and humans (e.g. cholera) [15].

The lack of a protocol for selection of community informants who ultimately detected and alerted MSF to public health threat was a design weakness of the system. Community discussions highlighted the need for more female key informants, and the benefit of having highly trusted community informants, both male and female, has been reported as a key CBS success factor [13]. One EBS evaluation in Vietnam reported that having a diversity of key informants likely enhanced the type of signals identified [12]. In contrast, most of our Tea Team key informants were male and this may have impacted the representativeness of the data collected and potential of the system to miss signals.

The system could have been improved by a defined and structured data-driven supportive supervision strategy, and documentation of its implementation, for community informants and CHWs.

Other strengths and weaknesses were identified linked to usefulness, PPV, and timeliness of signal verification. As above**,** the system produced useful data, but CIBS seemed to add little value overall and could be removed without having a big impact on utility. Multiple CIBS systems have been reported as producing useful public health data, but the reduced utility of the CIBS component in our system likely reflects the timelier and simpler approach of immediate reporting by phone that the CEBS approach provided or in part be due to the difficulty of implementing CIBS in semi-nomadic populations [11, 20]. CEBS systems have specifically been shown to produce useful data for public health action in a variety of low- and middle-income countries [5]. In the Tea Team surveillance system, there was variation in the usefulness of the signal types. For instance, there were few alerts and no responses conducted for AJS. AJS was included as a signal in the system due to an outbreak in 2017, however, this was not an ongoing risk, and AJS could have been removed as a priority signal [21]. Routine evaluation of the usefulness of signal types under surveillance is key to sustaining event-based surveillance [22]. Other evaluations of event-based surveillance systems have produced similar recommendations to drop signal types that have not proved useful in enabling early detection and response to outbreaks [12].

The overall PPV of a reported signal to the Tea Team surveillance system being an event was 14%, which falls in a similar range from other reported evaluations of community and health facility surveillance systems [5, 23]. Similarly to a previous study [23], event PPV varied by signal. The lower event PPV found in the CEBS component may reflect a more limited understanding of the signs and symptoms by community members and the need for regular refresher training of key informants and has been previously reported in other contexts [24]. The higher event PPV of the other event-based surveillance system reflects that these reports were from the RHB and other organizations and therefore had already undergone a degree of pre-verification.

Timely response to outbreaks is a core component of the Tea Team surveillance system and while most of the signals were verified within 24 h, there were often delays of more than 48 h when it came to risk assessment and response. These delays were typically due to operational challenges faced given the security and resources, outside of the influence of the surveillance system. The Tea team surveillance system was less timely compared to other community and health facility surveillance systems, for example, the mean time from detection to response in a community and health facility event-based surveillance system in Vietnam was 24 h [5]. Evaluation of an MSF health facility and community indicator-based system in Cox’s Bazar, Bangladesh also reported a more rapid triggering of the response mechanism compared to the Tea Team surveillance system [23]. However, Cox’s Bazar is a camp context with fewer logistical constraints in terms of organizing responses compared to the open context under surveillance in the Somali region.

This study had several limitations. We had incomplete documentation of why signals were excluded from the EWAR database and thus cannot be certain that the EWAR database fully reflects the public health threats that occurred in the zone during the evaluation period. Timeliness could not be fully assessed as the date of event occurrence was not systematically recorded in the EWAR database, preventing measurement of time from event to signal reporting. We did not fully evaluate acceptability of the surveillance system, as we did not conduct FGDs or interviews with CHWs or other surveillance stakeholders including the RHB and other actors who provided signals into the other event-based surveillance system. Due to the inconsistent documentation of health facility indicator-based signals into the EWAR database, we were not able to compare the functioning of the health facility surveillance component to the other components. Finally, the number of RHB-reported outbreaks was unavailable as a comparison metric; however, communities serviced by MSF had limited access to functional health facilities that document the bulk of outbreaks reported to RHB.

## Conclusions

Event-based surveillance approaches can produce useful data for public health action for pastoralist populations. The usefulness and acceptability of these surveillance systems can be further enhanced using participatory approaches in the design of these systems, such as agreeing on objectives, identifying appropriate signals, selection of key informants and design of clear community feedback mechanisms.

### Supplementary Information

**Additional file 1**.  Includes three supporting tables: (1) Case definitions to be used for the CIBS component of the Tea Team surveillance system, (2) Event definitions for the CEBS component of the Tea Team surveillance system, and (3) Roles and responsibilities in the Tea Team surveillance system.

## Data Availability

The datasets supporting the conclusions of this article are available on request in accordance with MSF’s data sharing policy. Requests for access to data should be made to oca.research@london.msf.org.
