# Supplementary material for: Evaluation of a multi-component early warning system for pastoralist populations in Doolo zone, Ethiopia: mixed-methods study
Source: Confl Health. 2024 Jan 30;18:13. doi: 10.1186/s13031-024-00571-y (PMC10829173; doi:10.1186/s13031-024-00571-y)
Supplement: Supplementary file 1 — Additional file 1. Includes three supporting tables: (1) Case definitions to be used for the CIBS component of the Tea Team surveillance system, (2) Event definitions for the CEBS component of the Tea Team surveillance system, and (3) Roles and responsibilities in the Tea Team surveillance system. [file 13031_2024_571_MOESM1_ESM.docx]

Annex

Table 1: Case definitions to be used for the CIBS component of the Tea Team surveillance system

| **Disease** | **Case definition**  **for Signals** | **Case definition**  **for verifying Signals and assessing Events** |
| --- | --- | --- |
|  | *To be used by: Local Informants, Community Health Workers* | *To be used by: Epidemiologist with Health Education Supervisor//Nurse team supervisor when deciding if a Signal is verified as an Event, and in RRT Assessment to decide if an Event should be confirmed as an Alert* |
| **Suspected Measles** | Adult or child with rash AND fever.  A cough, runny nose or red eyes makes measles more likely | One or more cases fitting the following case definition:  Fever ≥ 38^o^C (or verbal history of fever, not necessarily measured)  ***AND***  Presence of, or reported history of, generalised maculopapular rash (non‑vesicular)  ***AND***  Presence of, or reported history of, one of the following signs:   - cough - coryza (runny nose) - conjunctivitis (red eyes)   ***(OR:*** Any person a clinician suspects of having a measles infection) |
| **Suspected**  **Acute Jaundice Syndrome (AJS)** | Adult or child with new yellow colour in their eyes AND another symptom: fever, tummy pain, headache or other | One or more cases fitting the case definition:  An acute illness including acute jaundice, dark urine, anorexia, malaise/ extreme fatigue and right upper quadrant tenderness.  *[Source: OCA. Case definitions and epidemic thresholds of infectious diseases with epidemic potential. 2016]* |
| **Suspected**  **Acute Watery Diarrhoea (AWD)** | Adult or child with 3 or more liquid stools in the last 24 hours.  Signs of dehydration (sunken eyes, skin pinch slow to return, very weak and tired) and vomiting makes AWD more likely | One or more cases fitting the case definition:  ***Acute watery diarrhoea***: Adult or child with 3 or more WATERY (not just liquid) stools in the last 24 hours  **AND**  ***Signs of severe dehydration***:  - Any ONE of: coma, lethargy, pulse hard to palpate, very rapid breathing  *OR*  - TWO or more of: sunken eyes, skin pinch >2 seconds to disappear, patient drinks very little.  *[Source: MSF Cholera Guidelines. 2018, quoting from: Global Task Force on Cholera Control (GTFCC) Surveillance Working Group. Interim Guidance Document on Cholera Surveillance. June 2017]* |

Table 2: Event definitions for the CEBS component of the Tea Team surveillance system

| **‘Event’** | **Case definition**  **for Signals** | **Case definition**  **for verifying Signals and assessing Events** |
| --- | --- | --- |
|  | *To be used by: Local Informants, Community Health Workers* | *To be used by: Tea Team, PHO, RRT, and Mobile Clinic teams to verify signals AND during an assessment when deciding whether an Event classifies as an Alert* |
| **Cluster of similar illness** | Two or more cases of a similar illness occurring within 2 weeks  This can include a school reporting unusual absence due to similar signs and symptoms. | A greater than expected number of suspected cases of a moderate-to-severe illness within a 2-week period which could signify an outbreak or other crisis  *[Source: Local adaptation of: OCA, Case definitions and epidemic thresholds of infectious diseases with epidemic potential, 2016]* |
| **Increase in deaths** | More than the ‘normal’ number of deaths in the area in a 2-week period | A greater than expected number of deaths within a 2-week period which appear to be linked and which raise a concern of outbreak or other crisis (e.g. food insecurity, water shortage) |
| **Population movement** | Movement of 100HH or more into or out of the area | Movement of 50HH or more into a place lacking healthcare and/or water |
| **Concern about malnutrition** | Severe food shortage (for instance, people selling their last livestock for food or eating only 1 meal a day)  ***OR***  A death that might be due to malnutrition | Evidence of food shortage and/or poor animal health, combined with recent change in levels of malnutrition in children or PLW (from MUAC screening or admission rated to feeding programmes) |
| **Livestock illness or die‑off** | Illness or death in 5 or more animals | Illness or death from similar cause in 5 or more animals within a two-week period, which is outside of ‘normal’ and which could have implications for human populations |
| **Other unusual events or occurrences** | Any other event that you find unusual and concerning, that has an impact on health or healthcare, and that you think MSF should know about immediately (instead of waiting until a Weekly call)  *[Source: Technical Guidelines for Integrated Disease Surveillance and Response in African Region:*  *WHO and CDC 2010]* | Depends on the Signal – ask questions and use your judgement! |

Table 3: Roles and responsibilities in the Tea Team surveillance system

| **Role** | **Responsibilities in the Tea Team Surveillance System** |
| --- | --- |
| Local informants (volunteers) in Tea Team surveillance sites | - Gather information on defined ‘events’ (as in table 2) and report weekly to health education supervisor in phone call - Identify ‘events’ in the community requiring immediate alert and call within 24 hours to health education supervisor - Gather other community members to attend monthly community meetings with health education supervisor, to discuss health and healthcare issues and experience with MSF-OCA services |
| Community Health Worker (CHW) in MSF mobile clinic sites | - Gather information on defined ‘events’ (as in table 2) and report weekly to health education supervisor in phone call - Identify ‘events’ in the community requiring immediate alert and call within 24 hours to health education supervisor - Go door-to-door and collect household information on births, deaths, suspected AWD, measles and AJS occurring in their villages - Gather other community members to attend monthly community meetings with health education supervisor, to discuss health and healthcare issues and experience with MSF-OCA services |
| Health Education Supervisor (HES) | - Receive immediate alert calls from Tea Team volunteers and health facilities/MSF-OCA mobile clinics, gather and record, information, and report to epidemiologist - Receive weekly CHWs forms on numbers of suspected cases of AWD, measles and AJS as well as births, deaths, red MUAC, fast breathing and danger signs - Visit each Tea Team and CHW mobile clinic location each month to hold community meeting to discuss health and healthcare issues with community members - Provide refresher training to CHWs and community informants, as needed |
| Epidemiologist | - Develop and maintain required data collection forms, data collation tools and data visualisation tools for the Tea Team service - Train and maintain readiness of Rapid Response Teams - Discuss all Immediate alert calls received by health education supervisor, verify alert, if positive discuss with medical team lead and decide together the actions needed (alert assessment, rapid response team activation and intervention), enter details into EWAR database - Review weekly event-based and indicator-based surveillance data for alerts and general trends. If alerts/concerned identified, discuss with medical team lead and decide together the actions needed (alert assessment, rapid response team activation and intervention), enter details into EWAR database - Be part of alert assessment and/or Rapid Response Team as needed - Continually collect information on:   - population locations, size and movements   - Health facility functioning   - Mobile clinic and Tea Team locations - Review findings from monthly community meetings and discuss with medical team lead and Mobile Clinic Nurse Team Lead, along with mapping data, and alter services provision or locations of Mobile Clinics as indicated - Regular meetings with Regional health bureau and other actors to gather context information, share updates on Tea Team work |
| Nursing Team Supervisor (NTS) in mobile clinic teams | - Supervised the mobile clinic teams in collection and reporting of HFIBS data. - Provided supportive supervision to the CHWs, including on household visits and CIBS data collection and reporting |
| Rapid response teams | - Included epidemiologist, health educators, medical staff, and water/sanitation staff. - Conducted risk assessments for events to determine if the event is a health alert, to collect information needed to target a response, and to provide reactive responses during assessments. - Participated in MSF responses |
| Medical Team Leader (MTL) | - Decide with epidemiologist whether a risk assessment needed for a given event - Review risk assessment reports and other findings from rapid response teams and decide whether a response from MSF is needed or not - Coordinate the MSF response |
